# Report of Surgical Cases in the Buffalo General Hospital

**Published:** 1863-05

**Authors:** J. F. Miner


BUFFALO

### Medical and Surgical Journal

VOL. II. MAY, 1863. NO. 10.

### ORIGINAL COMMUNICATIONS.

ART. I.—*Report of Surgical Cases in the Buffalo General Hospital.* By J. F. Miner, M. D.

*Affections of the Uterus.*—Under this general head are grouped the various diseases of the os and cervix uteri, more definitely designated and generally known as granulation, ulceration, congestion, inflammation, or hypertrophy. Of these forms of disease we have nothing of especial interest to report; the cases treated were either connected with, or arose from syphilitic disease. The relative frequency of these affections in those who live dissolute lives is not readily determined, though leucorrhœa in some of its forms is believed to be generally present in those who indulge in inordinate and unnatural sexual intercourse. Examination often reveals the abraded, granular or inflamed surface from which it is secreted, though cases are quite common where the vaginal discharge is very profuse, and yet the diseased surface is beyond discovery. When it is furnished from the uterus or upper portion of the cervical canal, the os is usually large and patulous, and occupied by the tenacious stream of constantly escaping discharge, which is not easily detached or wiped away.

This class of patients rarely complain of other inconvenience than the discharge, and regard themselves cured when this is arrested, and diseased while it continues, consequently it is exceedingly difficult in many cases to discharge them satisfied of perfect recovery. The long list of sympathetic affections which are supposed to have their rise in granulations, ulcerations and congestions of the os and cervix uteri are for the most part entirely wanting. This fact is quite inconsistent with the view, that abrasions, congestions, ulcerations, &c. of the uterus invariably produce the various sympathetic derangements attributed to this cause, since the neck of the uterus is often extensively involved in disease, furnishing abundant secretion, and yet complaint is not made of any inconvenience other than the leucorrhœa.

Slight disease of the os or cervix uteri however often appears to exert a powerful influence upon the nervous system and a great variety of sympathetic affections will sometimes disappear when this irritation is removed. That this influence has been greatly over-estimated by some authors and practitioners, and as largely undervalued and depreciated by others, there can be no doubt. On the one hand, uterine irritation, granulation, congestion, &c. has been regarded as the ever-present, all-controlling source of disease and pain, and a deviation from the standard of perfect health regarded as uninvestigated, and its causes undiscovered until speculation had revealed its hidden source; while on the other, the more stupid and insensible position has been maintained, that uterine disease of this character is rare, its influence unimportant, and its careful investigation indecent and immoral. While the former view is to be avoided and guarded against, the latter is only to be regarded as the fruit of ignorance and jealousy.

Perhaps we should not dismiss this subject without a remark upon treatment, though we have only to confess to the adoption of a, perhaps, too common practice of cauterization. Irritable granulations upon the os or cervix uteri appear uniformly benefited by the application of the stick of nitrate of silver, provided it is not too frequently repeated. In the cases treated during the term of which report is made, no other local remedy was employed. In many cases of disease of the neck of the uterus, especially those of syphilitic origin, this practice seems attended by favorable results, if associated with proper constitutional treatment; yet universal and indiscriminate adoption of cauterization for all real and supposed disease of the neck of the uterus, is believed to be unnecessary, and often injurious; and though recovery may, and often does take place, it by no means indicates the usefulness of the medication. Reform of habits, rest, regulation of diet, and other circumstances of cleanliness and comfort, with proper general or specific treatment would prove curative in the same time, in many instances, without local medication.

*Paralysis.*—The single case of paralysis in the surgical ward was of long standing and produced by curvature of the spine. The patient when admitted was pale, thin and anæmic, but had at length gained his usual flesh and the appearance of general good health. The paralysis is so complete as to prevent the patient from walking except with the greatest difficulty, supported by two crutches. The attempt to relieve the pressure upon the spinal cord upon which the loss of power in the lower part of the body depends, has not yet been made. The case is still retained and offers opportunity for trial, but very little ground of expectation for favorable results.

*Syphilis and Gonorrhœa.*—The number under treatment for these diseases was proportionably large, since this is the only public institution in the city, where such disease is received for treatment. As it is not consistent with our purpose to describe cases so much singly, as in groups, we will only remark of the gonorrhœa that the rest, diet, and treatment of the hospital was successful in curing all cases in the shortest time consistent with the nature of the disease, cubeb and copaiba, constituting the staples of treatment. The single case of infecting chancre received treatment only in the hospital, while every patient with chancroid disease had been treated either by regular physician or quack, and by both alike so far as the administration of mercury is concerned; every case on admittance suffered more from mercurial ptyalism than from other cause. Many of the profession appear to be yet ignorant of the fact, that the simple chancre, which has for a long time been regarded as true syphilis, is now known to be an entirely distinct affection, completely local in its manifestations, and not requiring mercury in its treatment; those members of the profession who oftenest prescribe for these diseases in private practice are ignorant of this or hold to other and erroneous views. This fact has been demonstrated perhaps first by Bassereau, who by comparison of patients having venereal ulcers with the persons who infected them, has shown that when the disease remains local in the former, it was likewise so in the latter; and that if it affects the general system in the one, it has done so in the other; this result has also been confirmed by numerous observers. Besides this, clinical experience has as fully demonstrated it, and shown a wide difference between one class of cases, in which without the administration of mercury the disease forever disappears upon the healing of the ulcer, while in the other constitutional symptoms are sure to make their appearance, and often to re-appear during the life of the individual even under the best directed treatment. The soft chancre will generally heal spontaneously—certainly it cannot be hastened by administering mercury. The best treatment when seen early, is the destruction of the local sore by powerful caustic. Nitric acid is the agent most highly recommended. Nitrate of silver was used in the cases under treatment, since it was late before they came to the hospital, and the specific character of the sore was often changed before any application was made. Our treatment then consisted mainly in omitting medication, in these cases a very desirable measure, since the local sore and all other specific disease was absent, oftentimes before the mouth recovered from salivation.

These simple chancres gave rise to inflammatory bubo, a majority of which, terminated in suppuration. The abscesses were opened as soon as fluctuation could be detected, and tinc iodine, (where great indolence and inactivity was manifested,) injected into the cavity, compression being made, to hasten adhesion of the walls. In one case of great induration of the edges of the sore after opening the bubo, the whole hardened, indolent, unhealthy mass, was pared with a scalpel, which operation was attended by the best results, and an indolent, unhealthy sore of several weeks' standing was by this means immediately converted into a healthy granulating surface, which soon cicatrized completely.

Orchitis, iritis, and various secondary and tertiary forms of the disease were under treatment, and included with this class in our record, but since we desire to be brief in our report, and have nothing peculiar or unusual to note, we will omit both description and comment, except in cases included also under other diseases.

*Affections of the Eye.*—Syphilitic iritis, opake cornea, and granulated lids with opacity, comprise the list of diseases of the eye. The iritis had been under treatment for several weeks, disappearing and returning at irregular intervals. The treatment adopted for the inflammation of the iris consisted in dilatation of the pupil with atropia. The patient suffering at the same time from secondary symptoms required special medication, but for the relief of the iris nothing was required in addition to the atropia. Formerly, mercury was regarded essential in the management of this disease, and its influence supposed to be *plainly seen* through the "window open for the purpose," but it is now sufficiently demonstrated that this inflammation will subside spontaneously, and that if the curtain is drawn back to prevent adhesion, no injury to vision need be apprehended, certainly not in the great majority of instances, and if such result is feared from the severity of the inflammation, mercury will probably afford no protection whatever.

Several cases of granular lids and opacity of cornea were under treatment; they were old long-standing cases, which had been subject to violent medication for several years. Cupping, leeching, mercury, low diet, nitrate of silver, in strong solution and in solid stick, sulphate of copper, sulphate of zinc, scarification, &c., &c. had been tried until intolerance of light and almost total blindness was the condition to be remedied. Iodide of potassium in five grain doses, three times daily; generous diet, cleanliness, and *omission of all local medication* was followed by the most remarkable results. One patient who had been blind for two years was soon able to assist as dresser, and in others the improvement was not less, well marked. It may admit of some doubt which was most useful to these patients, the iodide of potassium or the omission of the local treatment, which had been conducted by various physicians and in different places, whither these patients had resorted for cure. Of the permanency of this improvement we cannot now speak positively, but from abundant observation, both in hospital and private practice, we have little hesitancy in opposing the long-continued application to the conjunctiva of concentrated solutions of nitrate of silver, sulphate of zinc, &c., &c. for the purpose of removing this granular condition, and of expressing confidence in the action of iodide of potassium, with generous diet, moderate use, and scrupulous cleanliness and attention to the general health; though we are not yet prepared to give a definite estimate of the separate value of the potassium.

*Laceration.*—The severest laceration was produced in one case by the Street Railroad Car passing over the leg and thigh; vessels were injured to such a degree that the cellular tissue soon filled with extravasated blood, and produced an immense slough of the integument extending around the thigh and nearly its whole length. Treatment consisted in allowing early and free escape of the extravasated blood, protection of the exposed surface by soft, warm poultice, relieving the pain when necessary with anodynes and sustaining the strength. Being a strong healthy man, he rallied from the shock, and is making progress towards recovery. If the view is correct that the skin looses its power of extending beyond certain limits, and that in consequence of this deficiency large ulcers cannot and do not heal without transplanting healthy skin to their centre from which it may extend, then in this case, skin will never cover the exposed surface without being re-enforced, for it is exceedingly large, and if the integument will ever in any case, cease to extend from failure of vital reproductive force this will almost certainly prove an example.

The second case under treatment was that of a young lad who was caught between a boat and the dock, removing integument and muscle from the outer portion of the leg. Granulations had supplied the place of the muscular tissue, and the skin had partly covered the surface, but gave no evidence of a disposition to extend and completely do so. At this stage of the treatment his friends removed him to his home, before we had opportunity to know positively what the the result might prove.

*Erysipelas.*—Only two cases of this disease appeared during the term of this report; these were in the ordinary form, appearing upon the face and scalp, and were not very severe in character. No effort was made to remove them from the ward, and no tendency was manifest to extend, both cases appeared to be idiopathic, and not a single case of traumatic erysipelas made its appearance.

The treatment recommended, was opium to relieve pain and procure sleep. Beef essence, and stimulants were administered, but the reliance was upon time, and the natural tendency of the disease to recovery.—Local applications were made by the house physician, Dr. Smith; but they were not very long continued, or regarded by him as more useful than flannel wrung from warm water and often applied. The disease is believed to be little influenced in duration or severity by medication, either general or local, and the principal objects of treatment to be, to allay pain, and support the strength, while the disease passes its usual period, which is not very uniform, or definitely fixed, and terminates in recovery.

Other classes of disease were recorded, but cases have been included often under different heads and considered sufficiently in connection with the associate affection, so that our brief report of groups of cases may be consistently completed without further consideration.

